# Gait speed associated factors in elderly subjects undergoing exams to obtain the driver’s license

**DOI:** 10.1590/1518-8345.2667-3138

**Published:** 2019-04-29

**Authors:** Maria Angélica Binotto, Maria Helena Lenardt, Nathalia Hammerschmidt Kolb Carneiro, Tânia Maria Lourenço, Clovis Cechinel, María del Carmen Rodríguez-Martínez

**Affiliations:** 1 Universidade Estadual do Centro-Oeste, Departamento de Educação Física, Irati, PR, Brasil.; 2 Universidade Federal do Paraná, Curitiba, PR, Brasil.; 3 Hospital Erasto Gaertner, Departamento de Enfermagem, Curitiba, PR, Brasil.; 4 Hospital de Clínicas, Unidade de Urgência e Emergência, Curitiba, PR, Brasil.; 5 Hospital do Idoso Zilda Arns, Curitiba, PR, Brasil.; 6 Universidade de Málaga, Departamento de Fisioterapia, Málaga, Espanha.

**Keywords:** Frail Elderly, Gait, Walking Speed, Automobile Driver Examination, Cross-sectional Studies, Aged, Idoso Fragilizado, Marcha, Velocidade de Caminhada, Exame para Habilitação de Motoristas, Estudos Transversais, Idoso, Anciano Frágil, Marcha, Velocidad al Caminar, Examen de Actitud para la Conducción de Vehículos, Estudios Transversales, Anciano

## Abstract

**Objective:**

to analyze the factors associated with gait speed in elderly subjects undergoing physical and mental fitness tests to obtain a driver’s license.

**Method:**

a cross-sectional quantitative study conducted in transit agencies. The probabilistic sample included 421 elderly (≥ 60 years old). The study was developed through application of questionnaires and tests that assess the frailty phenotype. For evaluating gait speed, the time spent by each participant to walk a 4.6 meter distance at normal pace on a flat surface was timed. Data were analyzed by using multiple linear regression and the stepwise method. The R statistical program version 3.4.0 was adopted.

**Results:**

there was a significant association between gait speed and paid work (<0.0000), body mass index (<0.0000), Mini-Mental State Examination (=0.0366), physical frailty (pre-frail =0.0063 and non-frail <0.0000), age (<0.0000), sex (=0.0255), and manual grip strength (<0.0000).

**Conclusion:**

elderly drivers who do not work, women of advanced age, high body mass index, low score in the Mini-Mental State Examination, low hand grip strength, and frail tend to decrease gait speed and should be a priority of interventions.

## Abstract (pt)

**Objetivo:**

analisar os fatores associados à velocidade da marcha em idosos submetidos aos exames de aptidão física e mental para habilitação veicular.

**Método:**

estudo quantitativo de corte transversal realizado nas clínicas de trânsito, no qual fizeram parte da amostra do tipo probabilística 421 idosos (≥ 60 anos). O estudo foi desenvolvido mediante aplicações de questionários e testes que constituem o fenótipo da fragilidade. Para avaliar a velocidade da marcha, cronometrou-se o tempo gasto pelo participante para percorrer uma distância de 4,6 metros, de maneira habitual e em superfície plana. Os dados foram analisados utilizando a regressão linear múltipla por meio do método *stepwise*. Adotou-se o programa estatístico R versão 3.4.0.

**Resultados:**

houve associação significativa entre velocidade da marcha e trabalho remunerado (<0,0000), índice de massa corporal (<0,0000), escore do Mini-Exame de Estado Mental (=0,0366), fragilidade física (pré-frágeis =0,0063 e não frágeis <0,0000), idade (<0,0000), sexo (=0,0255) e força de preensão manual (<0,0000).

**Conclusão:**

idosos motoristas que não trabalham, mulheres, com idade avançada, elevado índice de massa corporal, baixo escore no Mini-Exame de Estado Mental, baixa força de preensão manual e frágeis possuem tendência de diminuição da velocidade da marcha e devem ser prioridade das intervenções.

## Abstract (es)

**Objetivo:**

analizar los factores asociados a la velocidad de la marcha en adultos mayores sometidos a los exámenes de aptitud física y mental para habilitación vehicular.

**Método:**

estudio cuantitativo de corte transversal realizado en las clínicas de tránsito, en el cual hicieron parte de la muestra del tipo probabilística 421 adultos mayores (≥ 60 años). El estudio fue desarrollado mediante aplicaciones de cuestionarios y pruebas que constituyen el fenotipo de fragilidad. Para evaluar la velocidad de la marcha fue cronometrado el tiempo gasto por el participante para andar una distancia de 4,6 metros, de manera habitual y en una superficie plana. Los datos fueron analizados utilizándose la regresión linear múltiple por medio del método *stepwise*. Fue adoptado el programa estadístico R versión 3.4.0.

**Resultados:**

hubo una asociación significativa entre velocidad de la marcha y trabajo remunerado (<0,0000), índice de masa corporal (<0,0000), puntaje del Mini-Examen de Estado Mental (=0,0366), fragilidad física (pre-frágiles =0,0063 y no frágiles <0,0000), edad (<0,0000), sexo (=0,0255) y fuerza de prensión manual (<0,0000).

**Conclusión:**

adultos mayores conductores que no trabajan, mujeres, con edad avanzada, elevado índice de masa corporal, bajo puntaje en el Mini-Examen de Estado Mental, baja fuerza de prensión manual y frágil poseen tendencia de disminución de la velocidad de la marcha y deben ser prioridad de las intervenciones.

## Introduction

The autonomy, independence and mobility provided by vehicular driving are essential elements for the elderly’s well-being and quality of life^(^ ^1^ ^)^. The act of driving allows the access to different places and the performance of daily tasks, and these strengthen the satisfaction with life and the social bond.

Health conditions and functional declines associated with increasing age may affect the ability of driving a vehicle and this should be a concern of elderly drivers, their families, and transit and government agencies. Vehicle driving is a complex task involving motor, sensory and cognitive abilities that undergo age-related changes even in healthy aging conditions^(^ ^2^ ^)^, and such changes influence safe driving^(^ ^3^ ^)^.

Vehicle driving is a growing reality in this age group^(^ ^4^ ^)^. Statistics issued by transit agencies point to an increase in the number of elderly drivers. In 2005, the Brazilian National Transit Department registered 3.2 million drivers aged over 61 years, and in 2012, this number increased to 3.6 million^(^ ^5^ ^)^.

Given the conditions of elderly drivers and factors determining a safe transit, the main concern is the elderly in a disabling situation, particularly elderly individuals who already present some marker of physical frailty.

Physical frailty is “a medical syndrome with multiple causes characterized by decreased strength and endurance, reduced physiological functions that increase individuals’ vulnerability to development, and their dependency and/or death”^(^ ^6^ ^)^. It is associated with outcomes such as falls, dependency, hospitalization, institutionalization, death^(^ ^6^ ^-^ ^7^ ^)^, risk of limited recovery after illness, hospitalization or surgery and worse response to treatment^(^ ^8^ ^)^.

Functional aspects dependent on energy and speed of performance, and mobility-demanding tasks are affected by the frailty condition^(^ ^7^ ^)^. From this perspective, one of the markers of the frailty phenotype is reduced Gait Speed (GS). This is an indicator of the elderly’s health and well-being, and a powerful predictor of mortality^(^ ^9^ ^-^ ^10^ ^)^ associated with falls, cognitive impairment, functional incapacity, institutionalization^(^ ^11^ ^-^ ^12^ ^)^, old age, sedentary lifestyle and diseases^(^ ^13^ ^-^ ^14^ ^)^.

The greater number of elderly drivers and risks associated with driving a vehicle clearly demonstrate the need for regularly assessing the status of this activity by considering safety and the elderly’s capacity of continuing to drive^(^ ^4^ ^)^. According to the current traffic legislation^(^ ^15^ ^)^, the ability to drive does not address the elderly’s physical conditions, especially of the lower limbs, hence the GS is not measured.

The relevance of the study lies in identifying the factors associated with reduced GS for proposing and implementing preventive strategies directed to modifiable variables in order to assist the elderly with maintaining a safe vehicular driving. Knowledge about the theme may stimulate a new field of action for nursing. Gait speed has also been the target of studies involving elderly people in different contexts^(^ ^9^ ^-^ ^10^ ^)^ in spite of the knowledge shortage on this variable in relation to vehicular driving.

The aim of the present study is to analyze the factors associated with gait speed in elderly subjects undergoing physical and mental fitness tests for vehicular driving.

## Method

This is a cross-sectional quantitative study performed at transit agencies accredited for physical and mental fitness tests for vehicular driving.

For the sample calculation, was used the number of elderly (N) estimated by the Brazilian Institute of Geography and Statistics based on the last census, which was 198,089 elderly in the city where the study was developed. A 95% confidence interval (CI), significance level of 5%, 50% ratio estimation and 5% sample error were set. The final sample was of 384 elderly, to which were added 10% of losses and refusals possibilities. The final sample included 421 elderly.

The inclusion criteria were age ≥ 60 years, having scheduled and performed the physical and mental fitness tests for vehicular driving in one of the transit agencies. The exclusion criterion was to present temporary physical limitations for performing the tests (such as upper and/or lower limb fractures).

In total, 465 elderly people were invited to participate in the study, but 44 refused, so the sample included 421 elderly people.

The selection of transit agencies was through random sampling from an updated list (containing all agencies) provided by the Executive Transit Authority. The draw was processed manually and each agency represented a number from 1 to 54, because at the time of the survey (October 2014) there were 54 accredited agencies. All numbers (1 to 54) corresponding to the agencies were written in papers and mixed in an urn. The agencies were classified for data collection according to the draw order. Data from 35 elderly patients were collected at each agency, following the order of the agency draw until reaching the number of sample elements established for the study (n=421 elderly).

The distribution and scheduling of the elderly for undergoing physical and mental fitness tests at the transit agencies was performed through the Paraná Transit Authority system. From this equitable, random and unbiased distribution of the elderly, was determined the number of 35 elderly per agency in order to guarantee the homogeneity of data and reduce bias.

Fourteen agencies located in different neighborhoods in the city where the study was conducted were contacted in random order (defined previously). Two of these transit agencies were excluded because they did not have adequate physical space to perform the tests and the person in charge did not accept to participate in the study hence, 12 agencies were part of the study.

Data were collected from January 2015 to May 2016, and lasted approximately 30 minutes per participant. Before the start of data collection, the team of examiners (PhD students, Master’s students, and nursing undergraduate students linked to scientific initiation) was trained for standardizing the application of instruments and tests, and the form of approaching the elderly in the agencies. In addition, was conducted a pilot study with 15 elderly participants in order to adapt the collection instruments. Since there was no need for changes, the 15 subjects participating in the pilot study were included in the sample.

Data were collected through applications of questionnaires and tests. The structured questionnaire applied to the elderly included sociodemographic identification questions (age, sex, marital status, family organization, educational level, monthly income, race, income source: paid work, retirement, pensioner) and clinical information questions (health problems, falls, dizziness, fainting and vertigo, use of alcoholic beverages, use of tobacco, use of assistive technologies, use of medications, hospitalization, Body Mass Index -BMI)^(^ ^16^ ^)^.

The Mini-Mental State Examination (MMSE)^(^ ^17^ ^)^ was used for cognitive screening. The educational level was considered for the cut-off points^(^ ^17^ ^)^.

The following criteria were adopted to operationalize physical frailty^(^ ^7^ ^)^: self-report of fatigue/exhaustion, unintentional weight loss, decreased manual grip strength, reduced GS and decreased physical activity. Seniors with three or more of these characteristics were considered frail; those with one or two characteristics were pre-frail, and the elderly without any of these characteristics were considered as non-frail.

The evaluation of each physical frailty marker is described below. Fatigue/exhaustion was determined by self-reported answers to two questions of the Center for Epidemiological Scale-Depression, and all participants who marked ‘2’ or ‘3’ in any of the questions was classified as frail for this marker^(^ ^7^ ^)^. Unintentional weight loss was assessed by self-report, and any elderly who reported loss of body weight ≥ 4.5 kilograms in the last twelve months was considered frail for this marker^(^ ^7^ ^)^. Hand Grip Strength (HGS) was measured through a JAMAR^®^ hydraulic hand dynamometer. The average of three tests performed with the dominant hand squeezing to the maximum was considered as the final result. HGS values were adjusted by sex and BMI. The elderly in the lowest quintile (20%) were considered as frail for this marker^(^ ^7^ ^)^. For GS, was measured the time each participant took to walk 4.6 meters at normal gait on a flat surface. The final value was the average time spent to walk this distance for three times sequentially. After adjustment for sex and height, participants with GS values in the lowest quintile (20%) were considered frail for this marker^(^ ^7^ ^)^. Physical activity was determined by application of the Minnesota Leisure Time Activities Questionnaire. This instrument has been translated and adapted transculturally into Brazilian Portuguese^(^ ^18^ ^)^. This variable was adjusted for sex, and the elderly with values in the lowest quintile (20%) of caloric expenditure in physical activities were characterized as frail for this marker^(^ ^7^ ^)^.

In addition to GS, in this study, were evaluated the remaining markers of physical frailty, because the group of elderly individuals classified as frail, pre-frail and non-frail were variables of the study.

Data were inserted and coded into a Microsoft Excel spreadsheet, double-checked and information consistency was checked. Descriptive and inferential statistics were used for data analysis. Multiple linear regression with stepwise method was used to identify the variables associated with GS. The R statistical program version 3.4.0 was used, and GS was considered as a dependent variable. The results of regression analyzes were interpreted in terms of Odds Ratio (OR). Data were considered significant for p-values<0.05.

The research project was approved by the Ethics Committee on Research in Human Beings under number 833460. The ethical principles of voluntary and consensual participation were followed, because all elderly in this study signed the Informed Consent form, as stated in Resolution 466 of the National Health Council.

## Results

In the physical and mental fitness tests to obtain a driver’s license, the following predominated: male individuals (n=294; 69.8%), of white race (n=355; 84.3%), aged 60-69.9 years (n=278; 66.0%), married (n=288; 68.4%), tertiary educational level (n=160; 38%) living with the spouse (n=164; 39%), income of between 1.1 and 3 minimum wages (n=137; 32.5%) mainly from retirement (n=310; 73.6%) and paid work (n=217; 51.5%).

As for clinical characteristics, the following predominated: elderly with health problems (n=295; 70.1%), daily use of medications (n=280; 66.5%), BMI classified as eutrophic (n=225; 53.4%), no history of falls (n=382; 90.7%) and hospitalization in the previous 12 months (n=378; 89.8%), absence of dizziness, fainting or vertigo (n=409; 97.1%). In addition, elderly people who do not use assistive technologies (n=416; 98.8%), alcoholic beverages (n=329; 78.1%) and tobacco (n=379; 90.0%) also predominated.

Regarding the elderly’s physical frailty condition, 1.9% (n=8) were classified as frail, 44.9% (n=189) as pre-frail, and 53.2% (n=224) as non-frail. The prevalence of reduced GS as a marker of physical frailty was of 20.4% (n=86).

Table 1 shows the variables associated with GS in meters per second (m/s). The elderly’s condition of performing paid work increases GS by 0.0857 on average (p<0.0000; CI 95% [0.0453 - 0.12460]). Regarding the MMSE score, when increasing one unit, there was a GS increase of 0.0091 (p=0.0366; CI 95% [0.0005 - 0.0174]). For the covariable of physical frailty, in the transition from the frail to the pre-frail category, GS increases by an average of 0.2075 (p=0.0063; CI 95% [0.0591 - 0.3558]), while in the transition from frail to non-frail, GS increases by an average of 0.4334 (p<0.0000; CI 95% [0.2850 - 0.5817]). By increasing one unit of age, is expected a GS decrease of -0.0083 (p<0.0000; CI 95% [-0.0117 - -0.0049]). For the sex variable, men are on average 0.0722 faster than women (p=0.0255; CI 95% [0.0088 - 0.1356]). For each unit of increase in HGS, is expected an increase of 0.0100 in GS (p<0.0000; CI 95% [0.0067 - 0.0133]). BMI has a negative effect, so for each one-unit increase in BMI, is expected a decrease of 0.0126 in GS (p<0.0000; CI 95% [-0.01812 - -0.0071]).

**Table 1:** Results of multiple linear regression for variables associated with gait speed in the elderly. Curitiba, PR, Brazil, 2016

Gait speed (m/s)				

Covariable	Estimate	Standard Error	Z statistics	*p*-value*
Intercept	0.7531	0.1451	5.188	<0.0000
Paid work	0.0857	0.0206	4.145	**<0.0000**
MMSE score^†^	0.0091	0.0043	2.097	**0.0366**
Frail (Non-frail)	0.4334	0.0757	5.718	**<0.0000**
Frail (Pre-frail)	0.2075	0.0757	2.741	**0.0063**
Intercept	1.5803	0.1818	8.692	<0.0000
Age (years)	-0.0083	0.0017	-4.838	**<0.0000**
Sex	0.0722	0.0322	2.241	**0.0255**
HGS^‡^ (kgf^§^)	0.0100	0.0016	6.010	**<0.0000**
BMI^ǀǀ^ (kg/m^2¶^)	-0.0126	0.0027	-4.539	**<0.0000**

* p-value related to the regression coefficient of variables for each variable of the predictive model (significant for values<0.05); †MMSE - Mini-Mental State Examination; ‡HGS – Hand Grip Strength; §Kilogram/force; ǀǀBMI - Body Mass Index; ¶Kilogram per square meter

**Figure 1:**
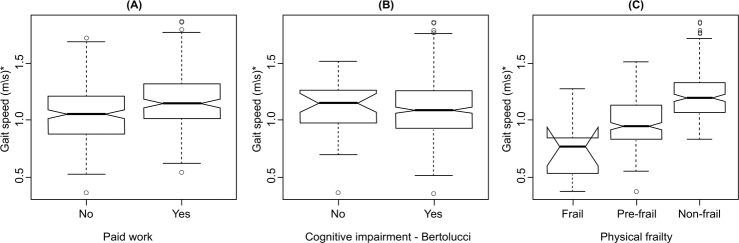
– Representation of variables of paid work (A), cognition (B), and physical frailty (C) for gait speed values of the elderly. Curitiba, PR, Brazil, 2016

Figure 1 shows the effects of the following variables: paid work, cognitive impairment and physical frailty in GS. The results corroborate the model adjustment by showing that working elderly (A), those with no cognitive impairment (B) and those classified as non-frail (C) presented higher values of GS with median values of 1.14 m/s, 1.15 m/s and 1.19 m/s, respectively.

*m/s - meters per second

**Figure 2:**
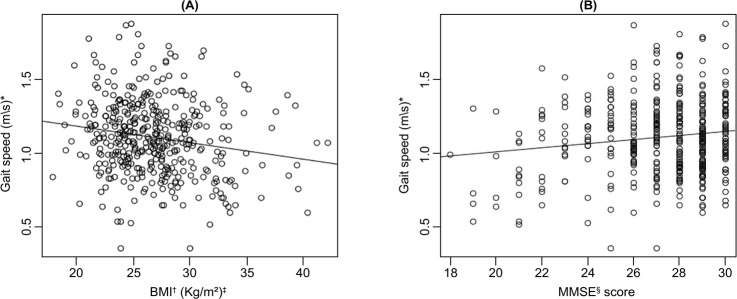
Representation of the values of Body Mass Index (A) and Mini-Mental State Examination score (B) for gait speed values in the elderly. Curitiba, PR, Brazil, 2016

The behavior of GS for BMI values and MMSE scores is shown in Figure 2. There is a tendency of GS decrease with increasing BMI values (A) with a correlation value of -0.1757 (-0.2668 | -0.0815) p=0.00029, and an increase in GS with increased MMSE scores (B) with a correlation value of 0.1372 (0.0422 | 0.2298) p=0.0047.

*m/s - meters per second; †BMI - Body Mass Index; ‡Kilogram per square meter; §MMSE - Mini-Mental State Examination

**Figure 3:**
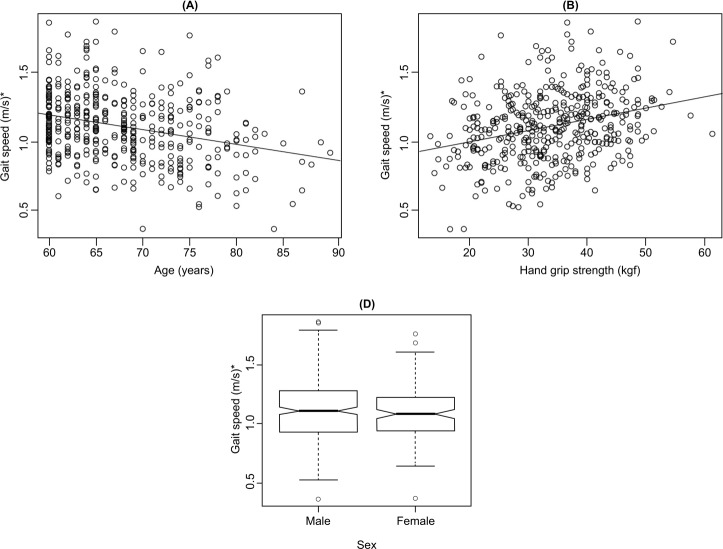
Representation of the variables age, hand grip strength and sex for gait speed values in the elderly. Curitiba, PR, Brazil, 2016

The behavior of GS according to age, HGS and sex is observed in Figure 3. There is a tendency that over the years, the elderly’s GS decreases (A), with a correlation value of -0.2852 (-0.3706 | -0.1499) p=2.53e-09. With increased hand grip strength, there is an increase in GS (B) with a correlation value of 0.2887 (0.1986 | 0.3739) p=1.58e-09. GS values are higher for men (median: 1.11 m/s) compared to women (median: 1.08) (D).

*m/s - meters per second

## Discussion

Reduced GS as a marker of frailty was present in 20.4% of the elderly who underwent physical and mental fitness tests to obtain a driver’s license. Similar percentages were found in a national study 20.9%^(^ ^19^ ^)^, and in an international study 21,9%^(^ ^20^ ^)^.

The variables significantly associated to GS were paid work, BMI, MMSE score, physical fragility, age, sex and HGS. Identifying this relationship between variables allows the proposition of interventions focused on modifiable variables.

The increase in GS related to the elderly’s paid work is explained in part by this being an active individual in society. However, it cannot be said that working keeps the elderly active or that they still work because they are active individuals. In general, working means better health conditions, and since GS is an indicator of health and well-being, data seem to reflect this positive influence of work in GS.

For the elderly, working is an important protection mechanism against depression and disability, helps to maintain well-being, good cognitive functioning and independence in activities of daily living^(^ ^21^ ^)^. Staying in the labor market is one of the proposals of the active aging policy. Working is one of the components of the participation pillar, an important element for social bonding, and associated with the elderly’s health and well-being^(^ ^22^ ^)^.

The relationship between BMI and GS reveals that increasing BMI values lead to a decrease in GS. This negative influence of BMI increase on GS values shows the unfavorable impact of overweight and obesity on the elderly’s physical function.

Studies are unanimous in recognizing that higher BMI values imply worse mobility and slower gait speed in the elderly. High BMI is associated with mobility limitation and poorer performance, as measured by GS (<1 m/s)^(^ ^23^ ^)^. High BMI values were associated with slow GS^(^ ^24^ ^)^. Furthermore, excessive adiposity also contributes to frailty, especially when it occurs together with decreased muscle mass and/or strength^(^ ^25^ ^)^.

As for cognitive impairment, with an increase in the MMSE score, there is an increase in GS. This finding demonstrates the positive effect of cognition on GS.

Results of studies conducted in other contexts found an association between GS and cognition. A study conducted in Curitiba/Brazil with 203 elderly (≥60 years) aimed to investigate the association between GS and the cognitive score of the elderly of a Basic Health Unit. There was a significant association between the cognitive score and GS (Prob>F=0.0072), and in direct proportion, the higher the cognitive score the greater the GS^(^ ^26^ ^)^. A prospective cohort study checked the relationship between GS and the incidence of dementia in community elderlies of three French cities (Bordeaux, Dijon and Montpellier). Participants were 3,663 elderly subjects (≥65 years) without dementia at the baseline followed for nine years. Slow gait speed was associated with increased risk of dementia (OR: 1.59; 95% CI 1.39-1.81; p<0.001) and gait was slower at seven years before the clinical onset of dementia^(^ ^27^ ^)^.

The association between slow GS and cognitive decline such as dementia is well documented in the scientific literature. A longitudinal study developed in the United States of America^(^ ^28^ ^)^ points to reduced GS as a factor that predates cognitive decline. This finding is especially important for directing preventive actions for this population, particularly elderly drivers.

The results for physical frailty demonstrated improvement in GS when the elderly passed from the frail to the pre-frail or non-frail condition. This effect was stronger for non-frail elderly compared to pre-frail elderly.

GS is one of the markers of physical frailty, since the functional aspects affected by the syndrome demand speed of performance^(^ ^7^ ^)^. GS is considered a predictor of frailty^(^ ^29^ ^)^, indicates physical decline, and is associated with the syndrome^(^ ^30^ ^)^.

Age had a negative effect on the elderly’s GS. This outcome indicates a trend that with each passing year the elderly become slower. The annual decline in GS was investigated in a longitudinal study with 2,364 elderly Americans from Memphis, Tennessee, Pittsburgh and Pennsylvania (mean age: 73.5±2.9 years, 52% women). The results showed that the group with GS decline had a decrease of 0.030 m/s per year (-0.028 - -0.033) or a relative decline of 21.7% over the eight-year period^(^ ^31^ ^)^. Preserving thigh muscle mass and preventing muscle fat infiltration are important aspects for decreasing age-related declines in GS^(^ ^32^ ^)^.

For the sex variable, men are on average faster than women. The gender difference in GS values is confirmed in other studies with higher mean values for men^(^ ^33^ ^-^ ^34^ ^)^.

The lower physical performance of women is explained by the distinct body structure of men and women. The lower physical function in women is explained predominantly by the greater amount of fat mass, but also by other differences in body composition^(^ ^35^ ^)^. Measures of basal adiposity are associated with a GS decline, especially in women^(^ ^32^ ^)^.

Data from the investigated elderly showed that muscular force positively influenced GS. By increasing HGS, there was an increase in GS. This finding shows a correlation between the variables, as confirmed in a study conducted in Hertfordshire/England. An association between HGS and the components of the Short Physical Performance Battery was found in a sample of 349 men and 280 women aged between 63-73 years. For men, the increase of one unit of HGS (JAMAR^®^ dynamometer) was associated with a decrease of 0.02 seconds in gait time (3 meters). In women, the increase of one unit of HGS was associated with a decrease of 0.03 seconds in gait time^(^ ^36^ ^)^.

The predictive power of HGS and leg extension strength in reduced GS (≤0.8 m/s) were compared with use of data from the Foundation of the National Institutes of Health Sarcopenia Project. A total of 6,766 elderly people aged 67 to 93 years participated in the project. The decrease in muscle strength defined by HGS was strongly associated with a greater chance of slow GS (OR: 1.99 to 4.33, c-statistics=0.53 to 0.67). An association between muscle weakness measured by grip strength and slow GS was found^(^ ^37^ ^)^.

Understanding the relationship between muscle strength and GS is relevant especially because they are interrelated with mobility, and consequently with aging people driving a vehicle. The elderly population mobility decline is closely linked to changes in the muscle strength-speed relationship^(^ ^38^ ^)^.

The driving license is necessary, and procedures for its issuance and renewal are varied. In Brazil, the current traffic legislation^(^ ^39^ ^)^ does not assign specific norms for the elderly, except for the shorter period (three years) for renewing the National Driver’s License from 65 years of age. In a study, funded by the ‘CONcerns and SOLutions - Road Safety in the Aging Societies’, the objective was to map and compare the licensing policy for vehicular driving in member states of the European Union. The conclusion reached was that European policies are coercive, not evidence-based, and susceptible to limiting the elderly’s mobility^(^ ^40^ ^)^.

At national level, the exams to obtain a driver’s license do not include tests focused on the lower limbs. This measurement becomes fundamental in elderly drivers given the decrease in muscle strength levels resulting from the aging process. Age-related degeneration of peripheral sensory receptors and nerves affect the lower limbs and the production of muscle strength, and lead to less precision in vehicular driving^(^ ^41^ ^-^ ^42^ ^)^.

The limitations presented by the study include the use of some data collection instruments with self-reported questions, which can generate bias. In addition, the instrument used to measure physical activity (Minnesota Leisure Time Activities Questionnaire) includes uncommon types of physical activity in the Brazilian context. Finally, the cross-sectional design does not allow determining the temporality of the analyzed factors.

Elderly subjects undergoing physical and mental fitness tests to obtain a driver’s license presented variables associated with GS that had been already identified in the literature, although in other contexts. Improving the modifiable factors may change the path of GS to a slower decline^(^ ^31^ ^)^. In addition, GS is susceptible to positive effects resulting from interventions. This aspect reinforces the relevance of identifying and proposing actions to elderly drivers with reduced GS. Improvement of physical functioning (GS and muscle strength) should be the focus of interventions for helping the elderly to maintain a safe driving^(^ ^43^ ^)^.

## Conclusion

The factors significantly associated with GS were paid work, BMI, MMSE score, physical frailty, age, sex and HGS. Elderly drivers who do not work, women of advanced age, high BMI, low MMSE score, low HGS, and frail have a tendency to decrease the GS. Interventions should be focused specifically on these groups in order to minimize and/or mitigate the decline in GS and thus, contribute to the safety of elderly drivers and those using the traffic routes.

The scientific literature shows that interventions involving physical exercise programs are effective for reducing body weight, improving muscular strength, GS and cognitive functions of the elderly. Joint actions/partnerships between transit agencies and the health system can facilitate the performance of a multidisciplinary team directed to the elderly with reduced GS. The involvement of health professionals is also necessary in discussions and proposals related to particularities of the aging process and the ability to drive motor vehicles. For gerontological nursing, the results provide subsidies for the implementation of actions directed to the elderly in the context of traffic.
